# The Clinical Significance of Imaging Biomarkers Discoverable by Anomaly Detection Methods in Retinal Diseases: A Review

**DOI:** 10.1155/joph/9792133

**Published:** 2026-05-05

**Authors:** Anna M. Wittmann, Philipp Seeböck, Katharina A. Heger, Daniel Egger, Georg Langs, Sebastian M. Waldstein

**Affiliations:** ^1^ Department of Ophthalmology, Landesklinikum Mistelbach-Gänserndorf, Mistelbach, Lower Austria, Austria; ^2^ Comprehensive Center for AI in Medicine, Medical University of Vienna, Vienna, Austria, meduniwien.ac.at; ^3^ Department of Biomedical Imaging and Image-Guided Therapy, Computation Imaging Research (CIR) Lab, Medical University of Vienna, Vienna, Austria, meduniwien.ac.at; ^4^ Department of Biomedical Imaging and Image-Guided Therapy, Computational Imaging Research (CIR) Lab, Medical Anomaly Detection (MANO) Group, Medical University of Vienna, Vienna, Austria, meduniwien.ac.at; ^5^ Salzburger Landeskliniken, Paracelsus Medical University Salzburg, Salzburg, Austria, pmu.ac.at; ^6^ Karl Landsteiner University of Health Sciences, Krems, Lower Austria, Austria, kl.ac.at

## Abstract

**Introduction:**

The increasing prevalence of vision‐threatening retinal diseases poses a growing challenge on healthcare systems worldwide, underscoring the need for effective strategies in disease diagnosis and management. Unsupervised artificial intelligence (AI) approaches, such as anomaly detection, offer the potential to identify established and novel retinal imaging biomarkers. They reduce reliance on expert‐driven annotations, mitigate bias, and overcome the limitations of predefined disease categories. Moreover, when integrated into clinical workflows, they have the potential to enhance practical applicability by supporting efficient screening, longitudinal disease monitoring, and objective assessment of treatment response.

**Areas Covered:**

This review examines the role of anomaly detection for discovering clinically relevant retinal imaging biomarkers, by categorizing biomarkers according to their underlying pathophysiology, summarizing the spectrum of anomaly detection methods applied in the field, and highlighting biomarkers that have been identified through these approaches.

**Critical Appraisal:**

Current anomaly detection methods favor the identification of established biomarkers characterized by strong intensity contrasts and well‐defined structural boundaries, while more subtle abnormalities remain difficult to capture. Future research should prioritize integrating large language models (LLMs), foundation methods, multimodality, and few‐shot anomaly detection, and increase explainability to advance the clinical application of anomaly detection for retinal imaging biomarker discovery in ophthalmology.

## 1. Introduction

The increasing prevalence of vision‐threatening retinal diseases imposes a growing burden on healthcare systems worldwide. Effective management of disorders such as age‐related macular degeneration (AMD), diabetic retinopathy (DR), and retinal vein occlusion (RVO) is particularly critical due to high patient numbers and resource constraints [1–3]. Imaging biomarkers are integral to disease management, serving not only as diagnostic tools but also as indicators for therapeutic decision‐making and monitoring of disease progression. The identification of novel imaging biomarkers is therefore essential to optimize patient outcomes. Nevertheless, the discovery of such biomarkers is often constrained by the necessity of expert knowledge, resource‐intensive manual analyses, and reliance on predefined categories of already established biomarkers. In this context, AI holds considerable promise for facilitating biomarker discovery and addressing these limitations [4–6].

Machine learning (ML), a subfield of AI, encompasses deep learning methods. They can be trained in a supervised manner, using datasets labeled by experts, or in an unsupervised manner, where the ML model identifies patterns directly from the data without manual annotation [7, 8]. Unsupervised approaches are particularly valuable in scenarios where labeled data are scarce. Among these, anomaly detection is a method to identify data points that deviate from the normal distribution observed during training. By highlighting unusual or unexpected imaging features, anomaly detection has the potential to uncover novel biomarkers that may be overlooked by conventional analysis, thereby reducing expert bias and overcoming the restrictions of current biomarker classifications [5, 6].

Despite the growing number of AI‐based studies in ophthalmology, to our knowledge, no comprehensive review has yet examined the role of anomaly detection for discovering clinically significant biomarkers in retinal imaging. This review addresses this gap by categorizing retinal imaging biomarkers according to their underlying pathophysiology, summarizing the spectrum of anomaly detection methods applied in the field, and highlighting biomarkers that have been identified through these approaches.

## 2. Clinical Background of Retinal Imaging Biomarkers

This section provides an overview of common retinal imaging biomarkers, with particular emphasis on their classification according to their underlying pathophysiological characteristics. Several imaging modalities are available to support biomarker identification in clinical practice. Among these, optical coherence tomography (OCT) has emerged as the gold standard diagnostic tool [9], complemented by techniques such as OCT angiography (OCT‐A), color fundus photography (CFP) [10], and fundus fluorescein angiography (FFA) [11]. OCT is particularly valued for its rapid noninvasive acquisition and its ability to generate high‐resolution cross‐sectional and volumetric images of retinal structures [12]. A focused light beam measures how different layers of the retina reflect light, producing a depth profile called an A‐scan. Sequential acquisition of multiple adjacent A‐scans produces a B‐scan, representing a two‐dimensional cross‐sectional image of the retinal layers. By further compiling a series of B‐scans across a defined region, a three‐dimensional volumetric dataset of the retina can be reconstructed [13]. This capability enables not only detailed structural visualization but also the quantitative assessment of clinically relevant biomarkers in retinal diseases [12]. Imaging biomarkers hold substantial predictive and prognostic value, underscoring the need for clinicians to understand the structural and functional features these biomarkers represent [14].

### 2.1. Retinal Swelling (Edema)

Macular edema occurs in various retinal diseases, which may result from distinct pathological processes. In order to quantify retinal thickness, central retinal thickness (CRT) is derived from retinal thickness maps and provides a reproducible quantitative means [15]. Accumulation of intraretinal or subretinal fluid, or the formation of cystoid spaces as in cystoid macular edema (CME), represents a common example and is a characteristic of DR, nAMD, and RVO [16–19]. Disruption of the blood–retina barrier allows excess fluid to accumulate within the intraretinal spaces, whereas impairment of the RPE and alteration of the choroidal vasculature promote fluid collection in the subretinal compartment. However, the pathophysiological mechanisms underlying intra‐ and subretinal fluid formation differ depending on the specific retinal disease [20].

In DR, fluid accumulation most commonly appears as intraretinal collection, characteristic of diabetic macular edema (DME) [21]. However, subretinal fluid can be present in DME as well, reflecting additional mechanisms such as RPE pump dysfunction and fluid overload, disruption of the ELM, and increased vascular endothelial growth factor (VEGF) expression, leading to higher vascular permeability [22–24]. In patients with RVO, macular edema typically is visualized as intraretinal macular fluid, with potential concomitant subretinal fluid collection due to elevated vascular permeability driven by increased expression of angiogenic factors [25–27]. In AMD, intra‐ and subretinal fluid can be seen independently on OCT images, occurring with or without the presence of macular neovascularization (MNV) [28]. The intra‐ and subretinal fluid accumulation in MNV differs by subtype and thus anatomical location of the neovascular membrane: MNV Type 1 is linked to subretinal fluid and frequently accompanied by fibrovascular pigment epithelial detachment (PED), Type 2 predominantly results in subretinal fluid, although intraretinal fluid may occur as well, and Type 3 is primarily associated with intraretinal fluid [16].

Accurate differentiation between subretinal fluid and PED is particularly important. A PED forms when the basal lamina of the RPE separates from the inner collagenous layer of Bruch’s membrane [16]. PEDs are categorized into three subtypes—serous, drusenoid, and fibrovascular—each demonstrating different characteristics on fundus examination, OCT, and FFA images [29]. PEDs most commonly occur in AMD [16]. In eyes with nAMD, chronic fibrovascular PEDs that have undergone repeated anti‐VEGF therapy may evolve into distinct multilayered PEDs, which appear on OCT imaging as organized layers of hyperreflective bands [30].

### 2.2. Atrophy

Retinal atrophy is characterized by tissue shrinkage due to decreased nutrition or as a consequence of trauma, leading to irreversible cell loss and areas of nonfunctional retina [31]. Both atrophy and layer‐specific degeneration are observed across a wide spectrum of retinal diseases, including, among others, AMD, DR, and RVO [31–33]. The loss of photoreceptor density, in particular, represents a hallmark feature of many inherited retinal diseases [34–36]. On OCT, atrophy of the important RPE layer manifests through hypertransmission of signal beneath the RPE that may extend into the choroid [31].

In AMD, atrophy has been further defined by the Classification of Atrophy Meeting (CAM) group [31] into complete RPE and outer retinal atrophy (cRORA), incomplete RPE and outer retinal atrophy (iRORA), complete outer retinal atrophy (cORA), and incomplete outer retinal atrophy (iORA). Each classification shows specific OCT‐based criteria. The group further proposed cRORA as the definitive endpoint of atrophy, while the commonly used term geographic atrophy (GA) should be reserved for end‐stage AMD without evidence of MNV, since cRORA can be present in nAMD as well.

Outer retinal tubulation (ORT) is characterized on OCT images by a hyporeflective lumen surrounded by a hyperreflective border, typically arranged in circular or ovoid configurations within the ONL [37]. ORT develops as a result of photoreceptor rearrangement following photoreceptor injury and RPE degeneration, as seen in nAMD, DR, and inherited diseases [37–40]. On OCT, ORT may mimic intra‐ or subretinal fluid. However, correct differentiation is crucial because ORT does not respond to anti‐VEGF therapy [41, 42].

Additionally, disorganization of the retinal layers can manifest on OCT images as alterations in the inner retinal layers, called DRIL [43], which is associated with reduced visual acuity in DR patients [44].

### 2.3. Fibrosis and Retinal Deposits

Fibrotic tissue typically appears as hyperreflective material on OCT images and represents a pathological repair process that arises after injury [45]. It is a common late‐stage manifestation in several retinal diseases, including AMD, DR, and central serous chorioretinopathy (CSC) [46–48]. In nAMD, fibrosis usually develops within the subretinal or sub‐RPE space, where it manifests as hyperreflective fibrous plates formed by fibrovascular tissue as part of MNV [49, 50]. In contrast, fibrosis in proliferative DR (PDR) typically arises in the preretinal or epiretinal layers as a part of fibrovascular proliferations [49, 51].

Drusen are extracellular deposits of lipids and proteins derived from the RPE that typically accumulate between the RPE and Bruch’s membrane and represent the hallmark feature of AMD. They are classified by size, with their volume being strongly associated with disease progression [52]. In addition to conventional drusen, other drusenoid deposits can be observed, called reticular pseudodrusen. They are located above the RPE in the subretinal space [53].

Hyperreflective foci (HF) represent small, round, backscattering lesions on OCT images that form within the neurosensory retinal layers [54, 55]. HF appear isolated or as clusters and have been identified in various retinal diseases, including AMD, DR, and RVO [56–59]. Notably, HF demonstrate a rapid response to anti‐VEGF therapy, with early changes detectable even before recurrent fluid accumulation, underscoring their role as sensitive biomarkers of disease activity [58, 60, 61]. Hard exudates represent a subtype of HF and can be found in AMD, DR, and RVO patients as well [45].

Additionally, hyperreflective deposits located between the neurosensory retina and the RPE can be visualized on OCT images as well, referred to as subretinal hyperreflective material (SHRM) [62]. It has been observed in several retinal and choroidal diseases [62–64] but plays a particularly important prognostic role in AMD patients, as its presence strongly correlates with neovascular activity [65].

### 2.4. Vascular Changes

Neovascularization represents the pathological growth of vascular and associated fibrous tissue. The most relevant forms can be categorized into neovascularization of the iris (NVI), neovascularization of the disc (NVD), and neovascularization elsewhere (NVE). They occur in a variety of ischemic ocular conditions, most prominently PDR, RVO, and ocular ischemic syndrome [66–69].

MNV refers to the growth of new blood vessels into the outer retina, subretinal, or sub‐RPE space and is commonly classified into three main types. In MNV Type 1, neovascular complexes arise from the choriocapillaris and proliferate through the dysfunctional Bruch’s membrane into the sub‐RPE space. In MNV Type 2, vessels penetrate the dysfunctional RPE and extend into the subretinal space. In MNV Type 3, neovascularization originates within the retina and extends into the subretinal or sub‐RPE space. MNV commonly induces exudation, resulting in intra‐ and subretinal fluid accumulation, hard exudate deposits, and SHRM due to blood–retina barrier breakdown and/or RPE pump dysfunction. MNV is most frequently associated with nAMD [16].

## 3. Anomaly Detection Methods as Tools for Retinal Biomarker Discovery

This section reviews anomaly detection methods with a focus on retinal imaging. Anomaly (or novelty) detection refers to the process of identifying data points that differ from the normal samples observed during training [5, 70]. These approaches are particularly valuable in scenarios where large amounts of normal data are available, but labeled examples of disease and rare abnormalities are scarce and difficult to obtain [70]. Depending on the algorithm, the output can be a global abnormality score or a segmentation map that localizes anomalous regions at the pixel or region level. The goal of anomaly segmentation is to precisely delineate pathological regions. This not only supports the detection of already established disease biomarkers in medical imaging but also holds the potential to uncover previously unknown biomarkers.

### 3.1. Fundamentals of Anomaly Detection

A variety of anomaly detection techniques have been developed, encompassing both traditional anomaly detection approaches and modern deep learning methods. Traditional and deep learning techniques can be applied independently or in combination [72]. Anomaly detection techniques can be grouped among others into the following categories: reconstruction‐based, density‐based, one‐class‐based, knowledge distillation–based (teacher–student), and vision–language model (VLM)–based methods.

**FIGURE 1:**
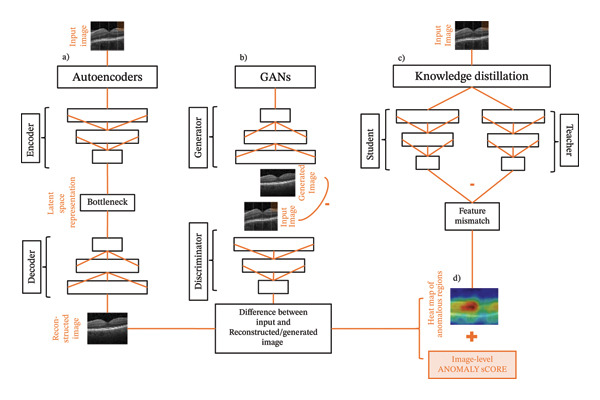
Main principles of autoencoders, GANs, and knowledge distillation. Images: [71]. Licensed under CC BY 4.0 (https://creativecommons.org/licenses/by/4.0/).

Reconstruction‐based approaches represent a key category in which models learn to reconstruct the normal input data during training. The underlying principle is that normal samples can be reconstructed accurately, while abnormal samples or regions result in higher reconstruction errors. The difference between the input and reconstructed image can therefore be used to detect anomalies. Beyond traditional ML approaches such as principal component analysis (PCA), deep learning–based anomaly detection models have been proposed, including methods based on autoencoders (AE) [73], variational autoencoders (VAE) [74], generative adversarial networks (GANs) [5, 73], or diffusion models [75–77]. As seen in Figure 1(a), an AE consists of two interconnected deep neural networks: an encoder and a decoder. The encoding network compresses the input data into a compact latent representation, and the decoding network transforms it back into the original data space [4]. In contrast, GANs (Figure 1(b)) consist of two components: a generator and a discriminator. Training is formulated as a zero‐sum approach, in which the generator seeks to produce synthetic images that are indistinguishable from real data, while the discriminator attempts to correctly differentiate between generated and authentic input data. Through this adversarial process, the generator progressively improves until it is capable of producing highly realistic synthetic output images [78].

Density‐based methods model the distribution of normal image features and flag samples that fall in low‐density regions as anomalous. The fundamental assumption is that by using the probability density function (PDF), normal data are mapped to high‐density regions of the distribution, whereas anomalous data occupy low‐density regions. In practice, modeling the full high‐dimensional image PDF is intractable, so modern pipelines estimate densities in a learned feature space. Closely related approaches are (1) classical density/probabilistic estimators, (2) normalizing flow (NF) methods that compute likelihoods via invertible mappings, and (3) memory bank/pretrained‐feature patch methods that approximate a local feature distribution via nearest neighbors or a compact memory. All three approaches produce patch‐ or image‐level anomaly scores and spatial heatmaps (Figure 1(d)) [70]. Density estimators include classic multivariate Gaussian and Gaussian mixture models [79], (robust) kernel density estimation [80], Bayesian methods [70], or combine deep learning with traditional PDF methods [79, 81]. In contrast, the underlying concept of NFs is to train a model that transforms images (or features) directly into a simple distribution through an invertible mapping. This allows direct computation of a log‐likelihood for each sample, where lower likelihood values indicate anomalies. Representative methods include DifferNet [82], CS‐Flow [83, 84], CFlow‐AD [85], or FastFlow [86]. Memory bank‐based methods use a pretrained network to compute embeddings for image patches. A representative set of normal patch embeddings is stored in a “memory bank.” New patches are then scored by computing their distance to the bank (e.g., nearest neighbor). Heatmaps are produced by aggregating patch scores. Representative methods include PatchCore [87], patch distribution modeling framework (PaDiM) [88], semantic pyramid anomaly detection (SPADE) [89], or pretrained anomaly detection adaptation (PANDA) [72].

One‐class methods aim to learn a boundary in feature space that encloses the normal training samples. During inference, the sample is projected into the feature space and detected as anomalous if lying outside of this boundary [71]. Representative approaches include one‐class support vector machine (SVMs) and support vector data description (SVDD) [90]. Multiple deep learning–based extensions have been developed, including DeepSVDD [91], PatchSVDD [92], DSPSVDD [93], or SE‐SVDD [94].

Knowledge distillation–based methods rely on a teacher–student framework (Figure 1(c)), in which the teacher’s network is often composed of a pretrained network, while the student network is trained exclusively on normal data. The main idea is that the student learns to mimic the teacher network. When presented with anomalous regions, however, discrepancies emerge between the representations generated by the teacher and those reproduced by the student, providing the basis for anomaly detection [95, 96]. Bergmann et al. [96] were the first to use a teacher–student architecture for anomaly detection. Additionally, multiresolution knowledge distillation (MKD) [95], student–teacher feature pyramid matching (STPM) [97], reconstruction STPM (RSTPM) [98, 99], reversed distillation (RD) [100], asymmetric student–teacher (AST) [101], and informative knowledge distillation (IKD) [102] approaches have been established.

VLMs are neural networks pretrained on large image‐text datasets to learn a joint feature space for image–text pairs and have recently been adapted for anomaly detection. In this setting, VLMs assess how well an image matches a textual description of “normal” anatomy or measure its similarity to prompts describing potential abnormalities. Approaches range from simple zero‐shot scoring with handcrafted prompts to learned prompt‐tuning and few‐shot in‐context adaptation. They operate at either image or patch level to produce both a global anomaly score and spatial heatmaps for localization. Representative recent works include WinCLIP [103], AnomalyCLIP [104], APRIL‐GAN [105], and AA‐CLIP (prompt learning and anomaly‐aware CLIP adaptations) [106], several CLIP‐based medical adaptations (MediCLIP [107], MedicalCLIP [107]), and exploratory pipelines that pair large VLMs with prompt or example conditioning [103–111].

Applications: In the medical domain, anomaly detection has been successfully applied to a wide range of imaging modalities, such as the identification of breast cancer in mammography [112], magnetic resonance imaging (MRI) [113] and ultrasound [114], lung lesions in chest radiographs [115], interstitial lung disease in computed tomography (CT) [116], findings in brain MRI [117], melanoma [118] and retinal pathologies in OCT [4–6, 71, 78, 119–125]. However, the applications of novelty detection are diverse and extend beyond medicine, including industrial inspection [84], intrusion detection systems [126], the analysis of videos [127], or hyperspectral remotely sensed imagery [128].

### 3.2. Anomaly Detection Methods in Retinal Imaging

In our view, the following studies represent the most relevant applications of anomaly detection in OCT imaging, encompassing AEs, GANs, Bayesian deep learning, pretrained feature networks, and knowledge distillation.

#### 3.2.1. AEs

Seeböck et al. [123] presented a novel unsupervised anomaly detection approach based on a deep convolutional autoencoder (DCAE) combined with a one‐class SVM to model the distribution of healthy OCT data. Through clustering, the resulting regions are segmented into subclasses. This method was the first to successfully identify abnormal regions in previously unseen images and to categorize these findings into distinct classes.

Building upon their previous work, Seeböck et al. [6] introduced an extended feature learning approach that provides a more comprehensive evaluation of anomaly detection in OCT images, identification of reproducible and stable categories, and the validation of potential biomarker candidates. The approach integrates a multiscale deep denoising autoencoder (DDAE) in combination with a one‐class SVM to robustly identify anomalies within the data. By using these marker candidates as classification features, they demonstrated a possible association between the detected markers and specific diseases.

Li et al. [129] presented SSL‐AnoVAE, a framework that integrates self‐supervised learning (SSL) with a VAE to enhance the extraction of prior semantic features. These features are combined with the original encoder representation, resulting in improved image reconstruction. The study further demonstrated the importance of data transformations in facilitating unsupervised anomaly detection. Moreover, the authors proposed an anomaly staging strategy based on residual clustering, which provides additional insights into retinal disease progression.

To address the limitations of anomaly detection performance, Zhou et al. [130] proposed a spatial–contextual VAE with attention correction (SCVAE‐AC). This approach enhances anomaly detection by increasing the separation between anomaly scores of normal and abnormal images. Furthermore, the authors introduced an ablation‐based method to compute anomaly attention maps in a gradient‐free manner, thereby improving interpretability.

#### 3.2.2. Generative Adversarial Networks

Schlegl et al. [5] introduced a novel framework based on DCGANs, called AnoGAN, marking the first application of GANs for anomaly detection overall. The trained DCGAN was employed to discriminate between normal and anomalous data. Furthermore, the authors proposed a mapping framework to better project images into latent space, improving the ability to differentiate samples that follow the training distribution from those that deviate.

Building on their previous work, Schlegl et al. [78] proposed a fast anomaly detection method based on a GAN, termed f‐AnoGAN. This unsupervised learning approach comprises two stages: First, a GAN is trained on normal OCT images; second, an encoder is trained using the pretrained GAN from the initial stage. Unlike their earlier work employing a DCGAN architecture [5], f‐AnoGAN utilized a WGAN backbone, although the framework is in principle adaptable to other GAN variants. The model enabled a direct mapping from input images to the learned latent space in a single inference step, making real‐time anomaly detection feasible. To train this encoder, the authors introduced a dedicated training phase guided by features from the pretrained discriminator, which replaced the slow iterative optimization used in their earlier work [5]. This approach substantially improved the speed of mapping images to the latent space and thereby enhanced the practicality of the proposed anomaly detection approach. It should be emphasized, however, that the model was evaluated on small‐scale OCT scans rather than the full‐width scans typically employed in clinical practice.

Liu et al. [124] extended the concepts of f‐AnoGAN and guided attention by proposing a novel hybrid model that integrated both approaches in a weakly supervised manner. Their framework incorporated an anomaly detection module and a classification network applied to OCT B‐scans. Unlike f‐AnoGAN, the proposed method processes the entire image as an input to preserve the topological consistency of the retinal layers. The model was trained in two stages. The anomaly detection network, composed of an encoder, a generator, and a discriminator is first optimized, after which the already trained discriminator is repurposed within the classification network as a classifier to reduce model complexity and generate a complete attention map. For precise biomarker detection, the authors combined normalized and refined attention maps and derived segmentation via a threshold operation. Notably, a simultaneous detection and classification of four already existing biomarkers was achieved through this approach.

#### 3.2.3. Bayesian Deep Learning

Seeböck et al. [125] introduced a novel anomaly detection framework that uses epistemic uncertainty estimates from a Bayesian U‐Net model to identify deviations from normal anatomy in new OCT image data. The model is trained using automatically generated labels rather than manual annotations, thereby constituting a weakly supervised anomaly detection strategy. In this framework, segmentation models were trained on healthy anatomy and MC dropout is applied to estimate epistemic uncertainty. The main assumption is that regions with higher uncertainty are more likely to represent abnormal anatomical structure.

Similarly, Mou et al. [131] presented an automated anomaly detection method based on epistemic uncertainty, introducing a Bayesian neural network model termed multiscale Bayesian U‐Net (MBU‐Net). Additionally, a threshold‐based function was employed to differentiate between healthy and anomalous OCT images.

#### 3.2.4. Pretrained‐Feature Frameworks

Tiosano et al. [71] evaluated several anomaly detection frameworks, all of which rely on pretrained CNN models, with the aim of identifying common retinal pathologies such as AMD, CNV, and DME. To adapt the pretrained features, the authors employed center loss adaptation for fine‐tuning. Specifically, they compared four previously introduced frameworks—SPADE, PANDA, PaDiM, and PatchCore—using established OCT datasets. Their approach consists of two steps: First, applying a pretrained CNN to datasets containing normal retinal features, and second, employing probabilistic models (kNN distance) to estimate the likelihood of local regions in new OCT scans. Regions with low likelihood scores were interpreted as abnormal structures, and scans containing one or more such regions were classified as anomalous. The generation of heat maps facilitated the visualization and assessment of each framework’s ability to localize anomalies within the images. Overall, this study highlights that pretrained feature extractors provide the basis for effective transfer of these frameworks to retinal OCT imaging, where they achieved consistently high area under the receiver operating characteristic curve (AUC–ROC) scores and successfully localized pathological regions associated with retinal diseases.

#### 3.2.5. Knowledge Distillation

Aresta et al. [12] introduced an unsupervised KD‐based deep learning framework for anomaly detection. An OCT scan was classified as anomalous if it exhibited signs of retinal diseases such as dry or wet AMD, GA, DME, CSC, RVO, or Stargardt disease. The method follows the reverse KD paradigm and integrates three CNN modules: a teacher, a student, and a bottleneck encoder. The model was trained on both volumetric and B‐scan levels, enabling robust anomaly detection. Beyond volume and B‐scan–level anomaly detection, the model also provides pixel‐wise anomaly maps.

Liu et al. [132] proposed TSSK‐Net, a weakly supervised framework for biomarker localization and segmentation that employs a teacher–student architecture with joint self‐supervised contrastive learning and knowledge distillation–based anomaly localization. Importantly, the method combines supervised contrastive loss with cross‐entropy loss to preserve anatomical structures while enhancing the feature encoding representations.

## 4. Retinal Biomarker Identification Through Anomaly Detection Methods

**FIGURE 2:**
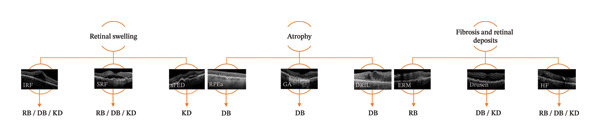
OCT biomarkers, their corresponding AD method used for identification, and their associated retinal pathophysiology. Abbreviations: serous PED (sPED), RPE atrophy (RPEa), reconstruction‐based (RB), density‐based (DB), knowledge distillation (KD). Clinical images from the authors’ own dataset, except image RPEa [133] and image DRIL [43]: Licensed under CC BY 4.0 (http://creativecommons.org/licenses/by/4.0/).

Image biomarkers can be identified using supervised and unsupervised methods. Supervised methods applied to OCT images include traditional supervised prediction [134], supervised deep learning for lesion segmentation [135], and outcome prediction using supervised deep learning [136], whereas unsupervised methods employed in OCT analysis encompass unsupervised feature learning [4] and anomaly detection. A wide range of retinal biomarkers have been identified using reconstruction‐, density‐, and knowledge distillation–based anomaly detection methods (Figure 2). Table 1 summarizes established retinal biomarkers in OCT images detected using these approaches and provides a structured overview linking anomaly detection methods to characteristic OCT patterns and their associated retinal disease contexts.

**TABLE 1:** Traditional retinal biomarker identification using anomaly detection methods.

Retinal pathophysiology	Imaging biomarker	Anomaly detection method	Network	Study
Retinal swelling	IRF	Reconstruction‐based	‐DCAE ‐DDAE ‐AnoGAN ‐f‐AnoGAN ‐WGAN and guided attention	Seeböck et al. [123] Seeböck et al. [6] Schlegl et al. [5] Schlegl et al. [78] Liu et al. [124] Seeböck et al. [125] Tiosano et al. [71] Aresta et al. [12]
		Density‐based	‐Bayesian U‐Net ‐SPADE, PANDA, PADiM, PatchCore	
		Knowledge distillation	‐Reverse KD	
	SRF	Reconstruction‐based	‐DCAE ‐DDAE ‐AnoGAN ‐f‐AnoGAN ‐WGAN and guided attention	Seeböck et al. [123] Seeböck et al. [6] Schlegl et al. [5] Schlegl et al. [78] Liu et al. [124] Tiosano et al. [71] Aresta et al. [12]
		Density‐based	‐SPADE, PANDA, PADiM, PatchCore	
		Knowledge distillation	‐Reverse KD	
	PED	Knowledge distillation	‐Reverse KD	Aresta et al. [12]

Atrophy	RPE atrophy	Density‐based	‐Bayesian U‐Net	Seeböck et al. [125]
	GA Lesions	Density‐based	‐Bayesian U‐Net	Seeböck et al. [125]
	DRIL	Density‐based	‐Bayesian U‐Net	Seeböck et al. [125]

Fibrosis and retinal deposits	ERM	Reconstruction‐based	‐WGAN and guided attention	Liu et al. [124]
	Drusen	Density‐based	‐Bayesian U‐Net ‐SPADE, PANDA, PADiM, PatchCore	Seeböck et al. [125] Tiosano et al. [71] Aresta et al. [12]
		Knowledge distillation	‐Reverse KD	
	HF	Reconstruction‐based	‐AnoGAN	Schlegl et al. [5] Seeböck et al. [125] Aresta et al. [12]
		Density‐based	‐Bayesian U‐Net	
		Knowledge distillation	‐Reverse KD	

Seeböck et al. [123] were able to identify clusters corresponding to the RPE, IRF, SRF, and the vitreoretinal border by employing a DCAE combined with a one‐class SVM. Building on this work, they later used a DDAE [6] coupled with a one‐class SVM to detect anomalies in OCT scans. This method successfully identified SRF, IRF, and pathological changes in the surrounding of the photoreceptor layer, as well as a novel biomarker category that could not be linked to specific retinal structures.

Using AnoGAN, Schlegl et al. [5] detected fluid accumulation, HF, and other anomalous regions without voxel‐level annotations. They subsequently introduced f‐AnoGAN [78], demonstrating improved accuracy in detecting fluid accumulation on the same dataset for comparability. Liu et al. [124] further advanced this approach by integrating f‐AnoGAN with guided attention in a hybrid weakly supervised model, enabling identification of OCT scans containing SRF, IRF, and ERM.

Seeböck et al. [125] also applied a Bayesian U‐Net to detect anomalies across multiple retinal diseases, including nAMD, GA, DME, and RVO. With this approach, they were able to successfully detect intraretinal cystoid fluid, HF, RPE atrophy GA lesions, drusen, DRIL, and cut‐edge artifacts. Tiosano et al. [71] evaluated pretrained CNN frameworks (SPADE, PANDA, PaDiM, and PatchCore) and demonstrated effective detection of IRF, SRF, and drusen.

Finally, Aresta et al. [12] employed a knowledge distillation–based approach using a reverse KD paradigm with three integrated CNN modules, enabling detection of retinal biomarkers, including IRF, SRF, PED, atrophy lesions, drusen, and HF.

As shown in Table 1, traditional retinal biomarkers can be detected using anomaly detection methods. However, a consistent observation is that these approaches perform best when applied to biomarkers with clear structural boundaries and pronounced intensity differences relative to the surrounding tissue. Well‐defined hyporeflective regions, such as IRF and SRF, and hyperreflective structures, such as DRIL, SHRM, ERM, HF, drusen, PED, and atrophy, were reliably identified across multiple studies [6, 12, 71, 78, 123–125]. Based on these findings, it seems reasonable to assume that anomaly detection could also be applied to other clearly delineated retinal biomarkers, including reticular pseudodrusen, pigmentary abnormalities, or fibrotic regions. In contrast, biomarkers with more subtle or less well‐defined OCT characteristics, such as reduced reflectivity of the EZ, might remain more difficult to detect, highlighting a current limitation of these methods. Addressing this challenge will require further research to enable the reliable identification of less distinct imaging features.

Using an unsupervised feature learning method based on a two‐stage AE architecture, Waldstein et al. [4] were able to identify a previously undescribed imaging feature characterized by punched‐out regions within the large choroidal vasculature, observed in patients with reduced visual acuity. In addition, the method successfully detected established biomarkers, including intraretinal cystoid fluid, SRF, SHRM, and PED. This method was designed to capture both local and global features in OCT scans of patients with AMD. The integration of such unsupervised feature learning strategies into future research may facilitate the discovery of additional novel retinal biomarkers.

## 5. Critical Appraisal and Future Perspective of Anomaly Detection

Supervised ML requires prelabeled data, making this paradigm both time‐consuming and costly. This limitation is particularly evident in medical imaging, where large, high‐quality annotated datasets are often scarce. Consequently, numerous studies on semisupervised and unsupervised anomaly detection methods have been successfully conducted. Notably, several studies suggest that semisupervised methods can outperform purely supervised approaches, particularly when combined with classical feature extraction, multiple instance learning (MIL)–based methods [137], GANs [138], or AEs [139].

However, one‐class and semisupervised approaches typically require large amounts of normal training data, which can be a limiting factor. Few‐shot anomaly detection offers a potential solution by enabling rapid model adaptation to new scenarios with minimal data from the target domain [84]. Recent work has explored VLMs in this context [103, 104, 110, 140], though further research is still needed to fully explore its applicability in the medical domain.

The black‐box nature of deep learning in anomaly detection underscores the need for explainability, as clinicians must understand why a region is flagged as anomalous for clinical decision‐making. Approaches such as feature attribution, attention maps, interpretably‐by‐design methods, and counterfactual explanations may increase transparency and interpretability. Additionally, reinforcement learning, human‐in‐the‐loop feedback, and multimodal integration can align model reasoning more closely with clinical decision‐making, indirectly supporting interpretability [141, 142].

Relying on a single imaging modality or data source risks overlooking critical functional aspects of disease. Consequently, multimodal strategies that combine imaging and nonimaging data are essential [84]. As certain biomarkers—such as MNV type or retinal ischemia—only emerge through cross‐modal relationships, expanding the use of multimodality in future research will be key to improving clinical applicability.

The emergence of large language models (LLMs) [143, 144] and foundation models [145] holds considerable promise for advancing anomaly detection in medical imaging. By leveraging pretrained representations, these approaches enable efficient learning from limited annotated datasets and support few‐shot detection of previously unseen anomalies. Furthermore, their ability to integrate multimodal data may help capture cross‐modal biomarkers and their relationships. Together, LLMs and foundation models have the potential to transform anomaly detection into a more data‐efficient, interpretable, and clinically relevant framework.

Publicly available datasets have facilitated the rapid development and evaluation of novel anomaly detection methods. Nonetheless, several challenges remain. Many datasets lack complete metadata, underscoring the need for standardized file formats and predefined data standards to enhance reproducibility. Furthermore, publicly available datasets often reflect ethnic and geographic biases, which may limit the generalizability of these models. To address these issues, ML paradigms such as domain adaptation or transfer learning offer promising strategies for improving applicability across diverse populations. Another limitation is disease coverage. Most publicly available datasets used for anomaly detection experiments predominantly focus on common retinal diseases such as DR and AMD, while other pathologies receive rather less attention [146].

Beyond biomarker discovery, anomaly detection has significant potential in clinical screening and disease monitoring. By identifying deviations from expected patterns, these methods might flag early signs of disease in at‐risk populations and track disease progression over time, enabling more timely and personalized interventions in the future. Additionally, integrating anomaly detection with other models, such as anomaly‐guided segmentation [147], can improve overall performance and facilitate clinical applicability.

## 6. Conclusion

In conclusion, current evidence demonstrates that anomaly detection methods can identify established retinal biomarkers in an unsupervised and unbiased manner, thereby reducing the reliance on labor‐intensive manual annotations by retina specialists. Nevertheless, these methods currently favor biomarkers with strong intensity contrasts and well‐defined structures, while less distinct abnormalities remain challenging to detect. Future work should focus on integrating LLMs, foundation methods, multimodality, and few‐shot anomaly detection to increase explainability and ensure the clinical applicability of anomaly detection in retinal biomarker discovery in the future. These approaches hold potential not only for detecting established biomarkers but also for discovering novel imaging features, ultimately supporting earlier diagnosis, personalized treatment, and improved patient outcomes. In particular, their integration into clinical workflows enhances practical applicability by supporting efficient screening, longitudinal disease monitoring, and objective assessment of treatment response.

## Author Contributions

Conception and design: Anna M. Wittmann, Philipp Seeböck, Sebastian M. Waldstein; writing–original draft preparation: Anna M. Wittmann, Philipp Seeböck, Sebastian M. Waldstein; writing–review and editing: Sebastian M. Waldstein, Georg Langs, Katharina A. Heger, Daniel Egger.

## Funding

No funding was received for this manuscript.

## Conflicts of Interest

Sebastian M. Waldstein reports consultancy for AbbVie, Bayer, Böhringer Ingelheim, DORC, Roche, and research support from AbbVie, Bayer, and Roche.

The other authors declare no conflicts of interest.

## Data Availability

The data that support these findings are openly available in databases.
